# Clinical Implications of Changes in Respiratory Instability Following Transcatheter Aortic Valve Replacement

**DOI:** 10.3390/jcm11010280

**Published:** 2022-01-05

**Authors:** Yohei Ueno, Teruhiko Imamura, Akira Oshima, Hiroshi Onoda, Ryuichi Ushijima, Mitsuo Sobajima, Nobuyuki Fukuda, Hiroshi Ueno, Koichiro Kinugawa

**Affiliations:** Second Department of Internal Medicine, University of Toyama, Toyama 9300194, Japan; fef6ge@gmail.com (Y.U.); brother0917jp@gmail.com (A.O.); ohiro0203@gmail.com (H.O.); ryuryu0702@gmail.com (R.U.); soba1126@yahoo.co.jp (M.S.); nfukuda@med.u-toyama.ac.jp (N.F.); hueno@med.u-toyama.ac.jp (H.U.); kinugawa-tky@umin.ac.jp (K.K.)

**Keywords:** heart failure, hemodynamics, sympathetic nerve activity

## Abstract

Background: Respiratory instability, which can be quantified using respiratory stability time (RST), is associated with the severity and prognostic impact of the disease in patients with chronic heart failure. However, its clinical implications in patients with severe aortic stenosis receiving transcatheter aortic valve replacement (TAVR) remain unknown. Methods: Patients who received TAVR and had paired measurements of RST at a baseline and one week following TAVR were prospectively included. Changes in RST following TAVR and its impact on post-TAVR heart failure readmissions were investigated. Results: Seventy-one patients (median age, 86 years old; 35% men) were included. The baseline RST was correlated with the severity of heart failure including elevated levels of plasma B-type natriuretic peptide (*p* < 0.05 for all). RST improved significantly following TAVR from 34 (26, 37) s to 36 (33, 38) s (*p* < 0.001). Post-TAVR lower RST (<33 s, *n* = 18) was associated with a higher 2-year cumulative incidence of heart failure readmission (21% vs. 8%, *p* = 0.039) with a hazard ratio of 5.47 (95% confidence interval 0.90–33.2). Conclusion: Overall, respiratory instability improved following TAVR. Persistent respiratory instability following TAVR was associated with heart failure recurrence.

## 1. Introduction

Aortic stenosis (AS) is a dominant cause of valvular heart diseases. Transcatheter aortic valve replacement (TAVR) is an established treatment of severe AS in a high surgical risk cohort [1] or even in a low surgical risk cohort [2,3]. The current concern is focusing on optimal patient selection to further improve clinical outcomes following TAVR.

The sympathetic nervous system is inappropriately activated during the progression of worsening heart failure [4]. Respiratory stability is largely affected by the inappropriate activation of the sympathetic nervous system via the association between the respiratory system, the autonomic centers, and hemodynamics [5,6,7]. Thus, respiratory instability is an established surrogate of heart failure severity. However, there has been no established methodology to accurately quantify the degree of respiratory instability.

Asanoi and colleagues recently introduced a novel methodology to quantify the severity of respiratory instability by measuring respiratory stability time (RST), which reflects the magnitude of the non-periodic irregular respiratory fluctuations as well as Cheyne–Stokes respiration [8]. All-night RST is a quantitative measure of respiration that reflects the clinical status of congestive signs and the recovery process from heart failure decompensation [9].

Patients with AS may also have respiratory instability [10] given their reduced cardiac output, increased sympathetic nerve activity [11], and concomitant sleep-disordered breathing. Respiratory instability may improve following TAVR due to hemodynamic amelioration [10]. Detailed analyses of peri-TAVR changes in respiratory instability may clarify the association between respiratory instability and AS and further help us risk-stratify TAVR candidates. In this study, we investigated peri-TAVR changes in RST as a primary concern and its prognostic impact as a secondary concern.

## 2. Methods

### 2.1. Patient Selection

Patients who received RST measurements two days before TAVR and one week after TAVR between the period of April 2017 to May 2020 were prospectively included. All patients had severe AS, which was defined as mean gradient ≥ 40 mmHg, peak velocity ≥ 4.0 m/s, and aortic valve area ≤ 1.0 cm^2^ (or ≤0.6 cm^2^/m^2^). This study was approved by the local institutional review board on 13 August 2018 (IRB 30-415). Written informed consent was obtained from each participant.

### 2.2. TAVR Procedure

The indication and detailed strategies for TAVR were determined by the heart-valve team consisting of board-certificated cardiologists, cardiovascular surgeons, and anesthesiologists. All patients received balloon-expandable valves (SAPIEN XT or SAPIEN 3; Edwards Lifesciences Inc., Irvine, CA, USA) or self-expandable valves (CoreValve or Evolut R; Medtronic, Minneapolis, MN, USA) under general or local anesthesia.

### 2.3. RST Measurement

**Figure 1:**
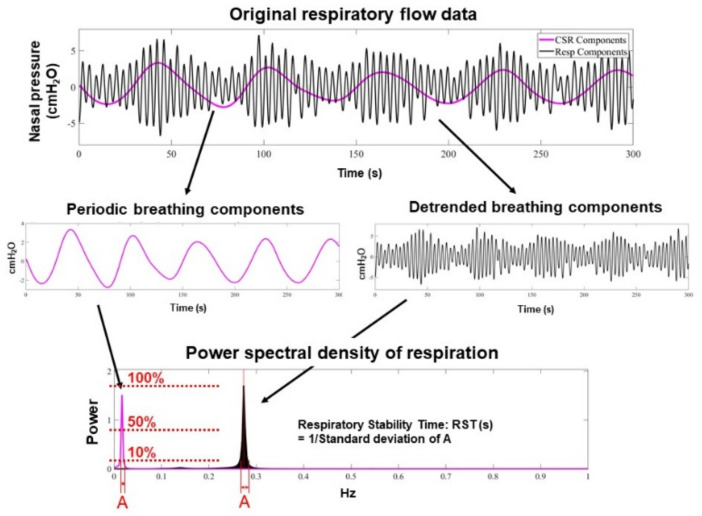
Methodology to calculate RST: All spectral power was normalized by the power spectral density or the ratio of the maximum power of the components. All respiration frequency points with a power spectral density > 10% were equally adopted in the assessment of respiratory instability. Very low frequency points of the periodic breathing curve were only adopted if the power spectral density of the very low frequency component was >50% of the maximum power of the respiratory component. Respiratory frequency points were evaluated using standard deviation, and RST was defined as the inverse of the standard deviation.

**Figure A1:**
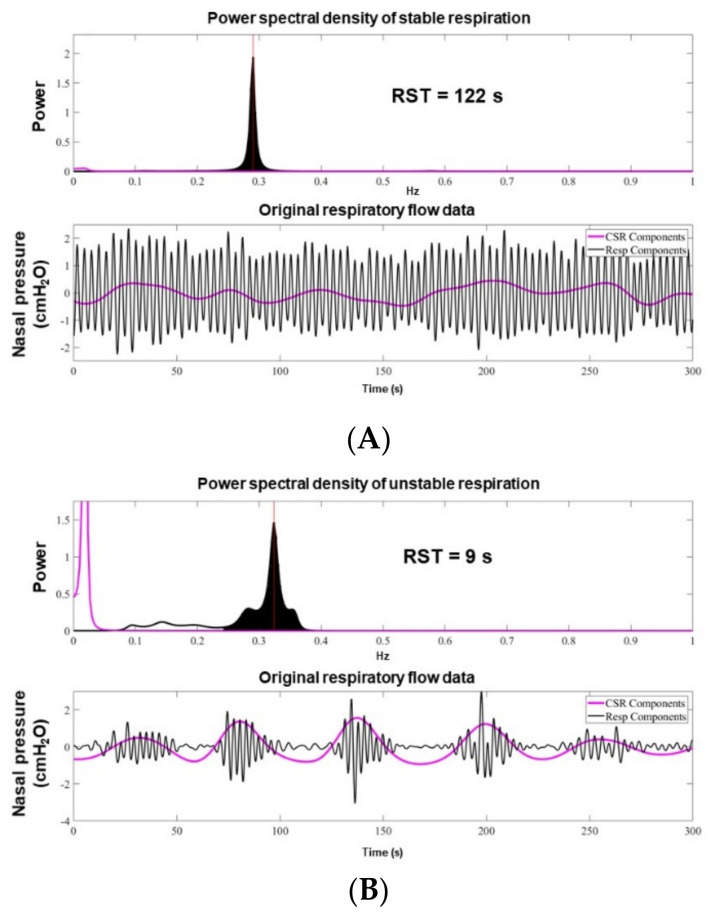
The representation of high RST (**A**) and low RST (**B**). In stable periodic breathing, the frequency spectrum is narrowly distributed and RST is high (**A**). In unstable periodic breathing, the spectral components are widely distributed and include very low-frequency components, resulting in a low RST value (**B**).

**Figure A2:**
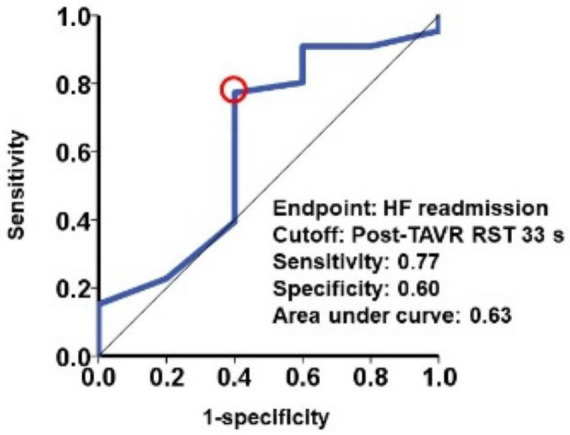
Cutoff of RST to predict heart failure readmission.

The methodology to measure RST about Figure 1 is detailed in Appendix A. In brief, the RST value is higher when the patient’s breathing patterns are stable and homogeneous during all-night monitoring. During stable periodic breathing, the frequency spectrum is narrowly distributed and RST is high (Figure A1). On the other hand, during unstable breathing, the spectral components are widely distributed and include very low frequency components, resulting in a low RST value (Figure A2).

### 2.4. Clinical Variables

Demographic, laboratory, hemodynamic, and echocardiographic data collected within one week prior to TAVR, as well as TAVR procedure data, were obtained as baseline characteristics. Post-TAVR laboratory and echocardiographic data were also obtained just prior to the index discharge. Heart failure readmissions that required intravenous diuretics during the 1-year observational period following the index discharge were also counted.

### 2.5. Statistical Analyses

Statistical analyses were performed using SPSS Statistics 22 (SPSS Inc., Armonk, IL, USA). Two-sided *p*-values < 0.05 were considered statistically significant. Continuous variables were expressed as the median and the interquartile. Categorical variables were expressed as the number and the percentage. RST levels tertiled by the several clinical variables were compared using the Kruskal–Wallis test. RST levels at the baseline and post-TAVR were compared using the Wilcoxon signed-rank test. A cutoff of post-TAVR RST to predict heart failure readmission was calculated using a time-dependent receiver operating characteristic analysis. The impact of the calculated cutoff of post-TAVR RST on the stratifying 2-year incidence of heart failure readmissions was investigated using the log-rank test and Cox proportional hazard ratio regression analysis. Multivariate analysis was not attempted, given the small event number. Change in RST following TAVR was a primary endpoint. The impact of post-TAVR RST on heart failure readmissions was a secondary endpoint.

## 3. Results

### 3.1. Baseline Characteristics

**Figure 2:**
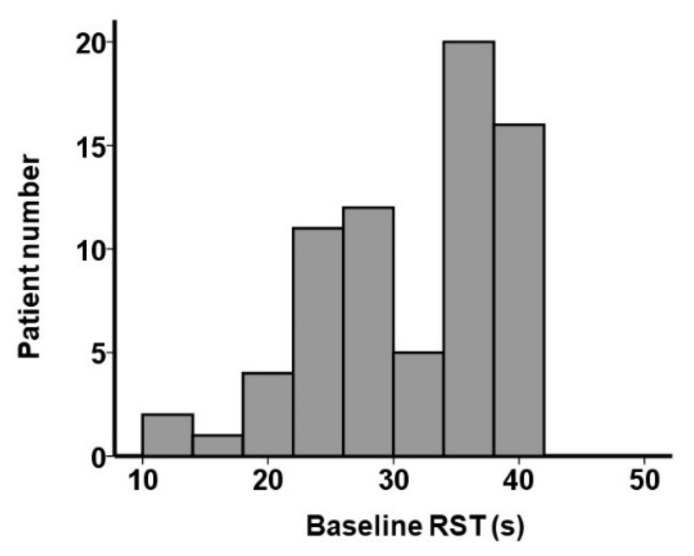
Distribution of baseline RST.

A total of 71 patients who received RST measurements before and after TAVR were included (Table 1). The median age was 86 (83, 88) years, and 25 (35%) patients were men. The maximum velocity at the aortic valve was 4.41 (3.99, 4.77) m/s, and the mean pressure gradient at the aortic valve was 46 (36, 55) mmHg. The Society of Thoracic Surgeons score was 4.6 (4.0, 6.7), and the EURO II score was 4.6 (3.6, 5.5). The baseline RST on the median was 34 (26, 37) s. The distribution of the baseline RST is displayed in Figure 2.

### 3.2. Baseline RST and Other Clinical Parameters

**Figure 3:**
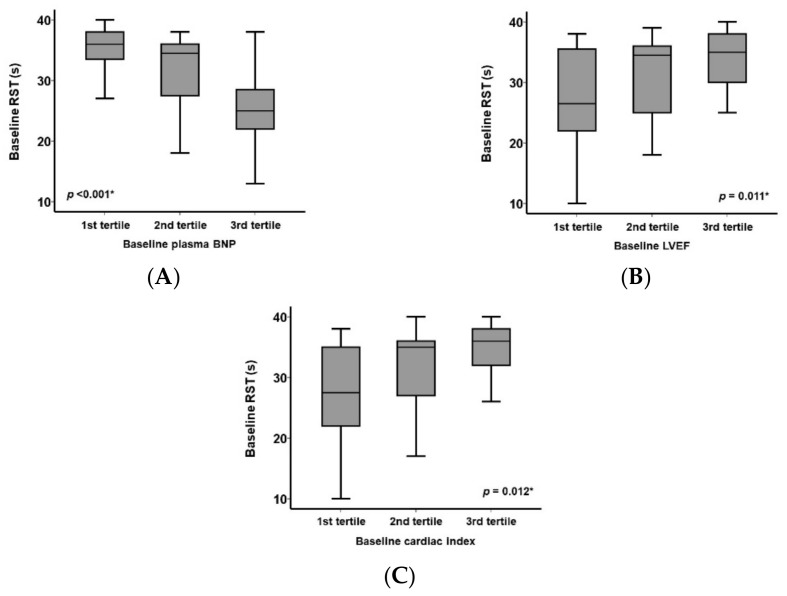
Baseline RST tertiled with baseline plasma B-type natriuretic peptide (**A**), left ventricular ejection fraction (**B**), and cardiac index (**C**). * *p* < 0.05 using the Kruskal–Wallis test.

The baseline RST showed clear correlations with several clinical variables associated with the severity of heart failure. A higher plasma B-type natriuretic peptide level was associated with a lower RST level (*p* < 0.001; Figure 3A). A lower left ventricular ejection fraction (*p* = 0.011) and cardiac index (*p* = 0.012) were associated with a decrease in RST (Figure 3B,C).

### 3.3. Change in Clinical Parameters Including RST Following TAVR

Following TAVR, the aortic valve area, the peak velocity at the aortic valve, and the mean pressure gradient through the aortic valve improved significantly (*p* < 0.01 for all). Changes in other clinical variables following TAVR are summarized in Table 2.

**Figure 4:**
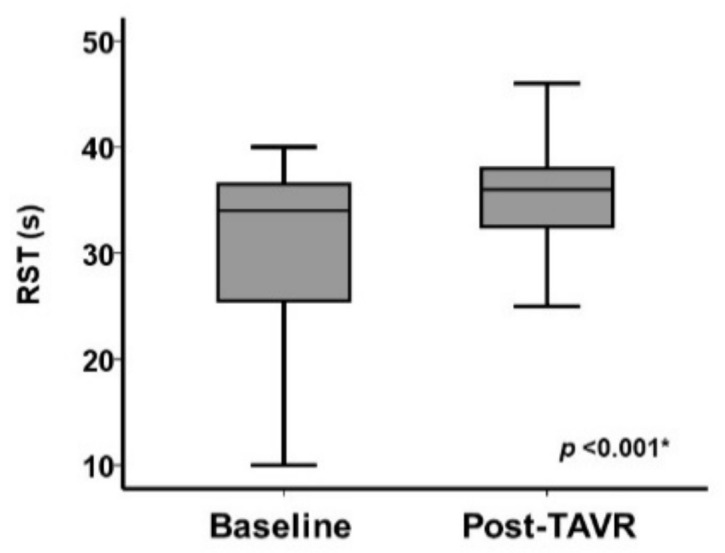
Change in RST following TAVR. * *p* < 0.05 using the Wilcoxon signed-rank test.

**Figure 5:**
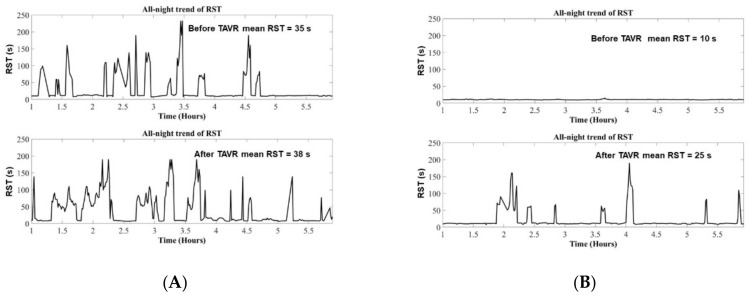
All-night trend of RST in a high RST case (**A**) and in a low RST case (**B**).

RST increased significantly following TAVR (from 34 (26, 37) s to 36 (33, 38) s, *p* < 0.001; Figure 4). All-night trends of RST examples before and after TAVR in patients with high RST (Figure 5A) and with abnormally low RST (Figure 5B) are displayed. Among 37/71 patients with baseline RST ≥ 33 s, RST remained high in most of them (36/37). Among 34/71 patients with baseline RST < 33 s, RST increased above 33 s in 17/34 patients and remained persistently low in 17/34 patients.

There were no significant differences in the baseline characteristics between those with post-TAVR low and high RST except for the higher plasma B-type natriuretic peptide level in the low RST group (Table 1).

### 3.4. Clinical Outcomes

There were no statistically significant differences in the occurrence of 30-day major events except for a trend in the higher incidence of heart failure in the post-TAVR low RST group (11% vs. 2%, *p* = 0.093; Table 3).

**Figure 6:**
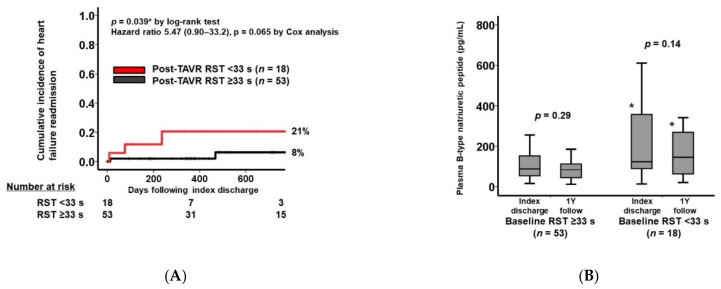
The cumulative incidence of heart failure readmissions stratified by the post-TAVR RST (**A**) and the trend in plasma B-type natriuretic peptide levels following index discharge (**B**) * *p* < 0.05 compared with the baseline RST ≥ 33 s group.

Following the index discharge, there were five heart failure readmissions during the 2-year observational period. Receiver operating characteristics analysis demonstrated a cutoff of 33 s for post-TAVR RST to predict the events with a sensitivity of 0.77 and a specificity of 0.60 (Figure A2). The cutoff of 33 s for RST significantly stratified the 2-year cumulative incidence of heart failure readmissions (21% in the low RST group vs. 8% in the high RST group, *p* = 0.039; Figure 6A). The hazard ratio of RST < 33 s was 5.47 (95% confidence interval 0.90–33.2) for the events (*p* = 0.065).

The plasma B-type natriuretic peptide level remained unchanged during the 1-year observational period irrespective of the RST level (*p* > 0.05; Figure 6B). However, the plasma B-type natriuretic peptide level remained significantly higher in the RST < 33 s group than the RST ≥ 33 s group at both the index discharge and at the 1-year follow-up (*p* < 0.05 for both).

## 4. Discussion

In this study, we investigated the change in RST following TAVR and the prognostic impact of post-TAVR RST. The major findings are as follows: (1) Baseline respiratory instability, which was indicated by the lower RST, was associated with more progressed heart failure in patients with severe AS; (2) RST increased significantly following TAVR in the entire cohort; (3) Post-TAVR RST < 33 s was associated with the incidence of heart failure admissions.

### 4.1. Respiratory Instability and AS

No previous studies have investigated the mechanism of respiratory instability in patients with AS, even though respiratory instability has a deep association with heart failure [5,6,7]. Given the various similarities between AS and heart failure, an underlying mechanism would be largely overlapped between heart failure and AS. The range of RST in clinically stable heart failure patients is reported between 20 s and 50 s [8]. Most of our patients also had comparable ranges of RST.

Sleep-disordered breathing stimulates sympathetic nerve activity and increases the afterload on the left ventricle in patients with heart failure, reducing their survival rate. Conversely, low cardiac output impairs peripheral circulation and increases bicarbonate chemo-sensitivity, leading to unstable respiration [12,13,14]. Pulmonary congestion stimulates vagal sensors in the lung, also deteriorating respiratory stability [15]. These factors might be enhanced, particularly in patients with advanced AS with impaired systemic circulation and elevated intra-cardiac pressure.

### 4.2. Impact of TAVR on Respiratory Instability

The detailed mechanism of why RST improved following TAVR in our overall cohort remains unknown. One of the explanations might come from the improvement in sympathetic nerve activity following TAVR that was demonstrated in previous studies investigating muscle sympathetic nerve activity [11] and cardiac metaiodobenzylguanidine scintigraphy [16]. TAVR results in afterload reduction on the left ventricle, as demonstrated in this study by the post-TAVR reduction in the plasma B-type natriuretic peptide level, improving vagal nerve activity and stabilizing respiratory instability. The wide distribution in the degree of RST improvement in each patient remains a future concern.

### 4.3. Respiratory Instability Following TAVR and Heart Failure Recurrence

Given the above-discussed deep association between respiratory instability and heart failure, it would be plausible that persistently low RST was associated with future heart failure occurrence following TAVR. Consistently, the PROST study demonstrated that RST was correlated with the degree of pulmonary congestion as well as the long-term prognosis in patients with heart failure [9]. Aggressive intervention in those with persistent respiratory instability following TAVR and its prognostic impact remains a future concern. For example, one promising therapeutic tool might be adaptive servo-ventilation, a non-invasive positive-pressure ventilation therapy that improves mortality and morbidity by reducing preload/afterload, suppressing sympathetic nerve activity, stabilizing respiratory abnormality, and increasing cardiac output in patients with congestive heart failure.

### 4.4. Limitations

First, this study is a proof of concept and included only a limited sample size. There is a scarcity of studies that validate the association between respiratory instability and RST in patients with AS. Further validation studies are warranted to expand the concept of RST in the AS cohort. Given the small event number, we could not perform multivariate analyses to adjust for potential confounders. We focused on heart failure recurrence as a clinical outcome, but the impact of RST on other outcomes remains unknown. RST remained low even after TAVR, and further specific strategies to improve RST following TAVR remains the future concern.

## 5. Conclusions

Overall respiratory instability improved following TAVR. Persistent respiratory instability, indicated by low RST, following TAVR was associated with heart failure occurrence.

## Figures and Tables

**Table 1:** Baseline characteristics.

	Total (*n* = 71)	Post-TAVR RST < 33 s (*n* = 18)	Post-TAVR RST ≥ 33 s (*n* = 53)	*p* Value
Demographics				
Age, years	86 (83, 88)	86 (83, 88)	85 (82, 88)	0.56
Men	25 (35%)	6 (33%)	19 (36%)	0.54
Body surface area, m^2^	1.45 (1.32, 1.57)	1.38 (1.30, 1.61)	1.47 (1.35, 1.58)	0.65
New York Heart Association (I/II/III/IV)	1/34/35/1	0/6/12/0	1/27/21/1	0.32
Comorbidity				
Diabetes mellitus	20 (28%)	4 (22%)	16 (30%)	0.37
Atrial fibrillation	5 (7%)	1 (6%)	4 (8%)	0.77
Ischemic heart disease	20 (28%)	7 (39%)	13 (25%)	0.19
Chronic obstructive pulmonary disease	4 (6%)	1 (6%)	3 (6%)	0.73
History of cardiac surgery	3 (4%)	2 (11%)	1 (2%)	0.16
Dyslipidemia	27 (38%)	6 (33%)	21 (40%)	0.41
Laboratory data				
Hemoglobin, g/dL	11.4 (10.4, 12.3)	11.5 (9.9, 12.4)	11.4 (10.4, 12.2)	0.99
Serum albumin, g/dL	3.8 (3.5, 3.9)	3.8 (3.6, 3.9)	3.7 (3.5, 3.9)	0.96
eGFR, mL/min/1.73 m^2^	51.2 (38.0, 60.2)	52.8 (36.8, 64.1)	51.2 (41.0, 60.2)	0.63
Serum sodium, mEq/L	141(139, 143)	140 (139, 141)	141 (139, 143)	0.12
Plasma B-type natriuretic peptide, pg/mL	245 (108, 495)	344 (191, 665)	258 (100, 476)	0.043 *
Aortic valve parameter				
Maximum velocity, m/s	4.41 (3.99, 4.77)	4.49 (3.83, 4.91)	4.31 (4.00, 4.73)	0.63
Mean pressure gradient, mmHg	46 (36, 55)	46 (34, 57)	45 (36, 53)	1.0
Valve area, cm^2^	0.59 (0.48, 0.69)	0.60 (0.52, 0.70)	0.59 (0.48, 0.67)	0.38
Echocardiography				
Left ventricular end-diastolic diameter, mm	44 (40, 50)	47 (44, 51)	48 (41, 54)	0.92
Left ventricular ejection fraction, %	66 (53, 73)	55 (53, 68)	65 (51, 73)	0.49
Moderate or greater mitral regurgitation	11 (15%)	5 (28%)	6 (11%)	0.095
Moderate or greater tricuspid regurgitation	1 (1%)	0 (0%)	1 (2%)	1.0
Hemodynamics				
Mean right atrial pressure, mmHg	6 (4, 8)	5 (4, 7)	6 (4, 8)	0.59
Pulmonary capillary wedge pressure, mmHg	13 (10, 16)	13 (10, 15)	13 (10, 16)	0.98
Cardiac index, L/min/m^2^	2.5 (2.3, 2.5)	2.5 (2.3, 2.8)	2.8 (2.4, 3.0)	0.11
Systolic blood pressure, mmHg	128 (118, 150)	112 (109, 134)	115 (104, 127)	0.95
Diastolic blood pressure, mmHg	63 (55, 76)	57 (51, 65)	56 (51, 67)	0.66
Heart rate, bpm	65 (60, 76)	66 (62, 75)	68 (59, 79)	0.64
Medication				
Beta-blocker	27 (38%)	9 (50%)	18 (34%)	0.18
Renin-angiotensin system inhibitor	45 (63%)	12 (67%)	33 (62%)	0.49
Scoring				
STS score	4.6 (4.0, 6.7)	5.2 (4.4, 7.3)	4.6 (4.0, 6.5)	0.39
EURO II score	4.6 (3.6, 5.5)	3.4 (2.3, 7.3)	4.6 (4.0, 6.5)	0.79
RST, s	34 (26, 37)	28 (25, 30)	38 (36, 39)	<0.001 *

eGFR, estimated glomerular filtration ratio; RST, respiratory stability time. * *p* < 0.05 using the Wilcoxon signed-rank test.

**Table 2:** Peri-procedural clinical parameters.

	Baseline	After TAVR	*p* Value
Laboratory data			
Hemoglobin, g/dL	11.4 (10.4, 12.3)	10.4 (9.8, 11.6)	<0.001 *
Serum albumin, g/dL	3.8 (3.5, 3.9)	3.4 (3.1, 3.6)	<0.001 *
eGFR, mL/min/1.73 m^2^	51.2 (38.0, 60.2)	54.5 (40.6, 64.6)	0.30
Serum sodium, mEq/L	141 (139, 143)	139 (137, 141)	<0.001 *
Plasma B-type natriuretic peptide, pg/mL	244.5 (107.5, 495.1)	103.4 (55.1, 187.4)	<0.001 *
Aortic valve parameter			
Maximum velocity, m/s	4.41(3.99, 4.77)	1.98 (1.59, 2.32)	<0.001 *
Mean pressure gradient, mmHg	46 (36, 55)	8 (5, 11)	<0.001 *
Valve area, cm^2^	0.59 (0.48, 0.69)	1.48 (1.24, 1.65)	<0.001 *
Echocardiography			
Left ventricular end-diastolic diameter, mm	44 (40, 50)	43 (37, 49)	0.40
Left ventricular ejection fraction, %	66 (53, 73)	64 (55, 77)	0.81

eGFR, estimated glomerular filtration ratio. * *p* < 0.05 using the Wilcoxon signed-rank test.

**Table 3:** Post-TAVR 30-day major events.

	Post-TAVR RST < 33 s (*n* = 18)	Post-TAVR RST ≥ 33 s (*n* = 53)	*p* Value
Total major events	4 (22%)	8 (15%)	0.49
Permanent pacemaker implantation	2 (11%)	5 (9%)	0.84
Heart failure	2 (11%)	1 (2%)	0.093
Major bleeding	0 (0%)	1 (2%)	0.56
Stroke	0 (0%)	1 (2%)	0.56

## Data Availability

Data are available from the corresponding authors upon reasonable requests.

## Appendix A

All-night respiratory measurements were performed using a respiratory monitoring device (Morpheus R, Teijin, Tokyo, Japan). Air flow was measured using a nasal cannula with a pressure sensor, and arterial oxyhemoglobin saturation (SpO_2_) was also continuously measured with a pulse oximeter (Morpheus R, Teijin, Tokyo, Japan).

As summarized in Figure 1, all-night respiratory nasal pressure signals were digitized and sampled at 32 Hz. The data to be included in the analysis were restricted to those for the fixed hours from 23:00 through 05:00 h. These respiratory signals were resampled at 4 Hz and integrated to obtain instantaneous ventilatory volume. To serially calculate all-night RST, the respiratory signals were divided into serial 5 min segments every 50 s. More than 420 segments of 5 min data were analyzed. We focused on two frequency ranges to estimate RST. One range consisted of respiratory frequency components obtained from the instantaneous ventilation signal with high- or low-frequency noise removed via a 5th order Butterworth bandpass filter with cutoff frequencies of 0.11 Hz and 0.5 Hz. Other components constituted a very low-frequency range of respiration as an indicator of periodic respiration magnitude. These data were obtained by adjusting the baseline to zero, tracing the peak of the instantaneous ventilation signal, and applying a bandpass filter with cutoff frequencies of 0.008 and 0.04 Hz. For each 5 min epoch, the maximum entropy method was applied to these respiratory and periodic breathing curves to extract the specific components of respiratory variations from each wave.

We applied MEM to these respiratory and periodic respiratory curves and extracted the spectral components of respiratory variability from each wave measured (Figure 1). All spectral power was normalized by the power spectral density (PSD), or the ratio of the maximum power of the components. This describes how the power of a signal or time series is distributed over frequency. All respiration frequency points with PSD > 10% were equally adopted in the assessment of respiratory instability. Very low frequency points of the periodic breathing curve were also adopted only if the PSD of the very low frequency component was >50% of the maximum power of the respiratory component.

Respiratory frequency points were evaluated using standard deviation, and RST was defined as the inverse of the standard deviation. The RST throughout the night was averaged as a measure of all-night respiratory stability. Since RST is the inverse of frequency (Hz), it is expressed as a unit of time (s). In other words, it corresponds to the time during which the unchanged and stable breathing pattern is repeated and indicates the stability of breathing.
